# Predictors of failed intrauterine balloon tamponade for persistent postpartum hemorrhage after vaginal delivery

**DOI:** 10.1371/journal.pone.0206663

**Published:** 2018-10-26

**Authors:** Joséphine Grange, Manon Chatellier, Marie-Thérèse Chevé, Anne Paumier, Claudine Launay-Bourillon, Guillaume Legendre, Marion Olivier, Guillaume Ducarme

**Affiliations:** 1 Department of Obstetrics and Gynecology, Centre Hospitalier Départemental, La Roche sur Yon, France; 2 Réseau Sécurité Naissance des Pays de la Loire, Nantes, France; 3 Department of Obstetrics and Gynecology, Le Mans General Hospital, Le Mans, France; 4 Department of Obstetrics and Gynecology, Atlantic Polyclinic, Saint-Herblain, France; 5 Department of Obstetrics and Gynecology, Jules Verne Clinic, Nantes, France; 6 Department of Obstetrics and Gynecology, Angers University Hospital, Angers, France; Case Western Reserve University, UNITED STATES

## Abstract

**Objective:**

To identify the predictors of intrauterine balloon tamponade (IUBT) failure for persistent postpartum hemorrhage (PPH) after vaginal delivery.

**Design:**

Retrospective case-series in five maternity units in a perinatal network.

**Setting:**

All women who underwent IUBT for persistent PPH after vaginal delivery from January 2011 to December 2015 in these hospitals.

**Methods:**

All maternity apply the same management policy for PPH. IUBT, using a Bakri balloon, was used as a second line therapy for persistent PPH after failure of bimanual uterine massage and uterotonics to stop bleeding after vaginal delivery. Women who required another second line therapy (embolization or surgical procedures) to stop bleeding after IUBT were defined as cases, and women whom IUBT stopped bleeding were defined as control group. We determined independent predictors for failed IUBT using multiple regression and adjusting for demographics with adjusted odds ratios (aORs) and 95% confidence intervals (95% CI).

**Results:**

During the study period, there were 91,880 deliveries in the five hospitals and IUBT was used in 108 women to control bleeding. The success rate was 74.1% (80/108). In 28 women, invasive procedures were required (19 embolization and 9 surgical procedures with 5 peripartum hysterectomies). Women with failed IUBT were more often obese (25.9% vs. 8.1%; p = 0.03), duration of labor was shorter (363.9 min vs. 549.7min; p = 0.04), and major PPH (≥1,500 mL) before IUBT was more frequent (64% vs. 40%; p = 0.04). Obesity was a predictive factor of failed IUBT (aOR 4.40, 95% CI 1.06–18.31). Major PPH before IUBT seemed to be another predictor of failure (aOR 1.001, 95% CI 1.000–1.002), but our result did not reach statistical significativity.

**Conclusion:**

Intrauterine balloon tamponade is an effective second line therapy for persistent primary PPH after vaginal delivery. Pre-pregnancy obesity is a risk factor of IUBT failure.

## Introduction

Postpartum hemorrhage (PPH) constitutes a major component of severe maternal morbidity and mortality and complicates approximately 5% to 15% of all deliveries [1]. After failure of primary management of PPH after vaginal delivery, second-line treatments, such as pelvic arterial embolization, vessel ligation, uterine compression sutures, can be attempted to achieve arrest of severe persistent PPH, defined as excessive bleeding (1,000 mL or greater) within the first 24 hours after birth [2], and to avoid peripartum hysterectomy [3–6]. In a recent large population-based retrospective cohort study included 72,529 women in two French perinatal networks, invasive procedures (pelvic vessel ligation, arterial embolization, hysterectomy) were used in 4.1 per 1,000 deliveries [7].

Recently, a summary of studies showed that intrauterine balloon tamponade (IUBT), as Bakri balloon (Cook Medical, Bloomington, IN, USA), is an effective tool to avoid invasive procedures treating persistent PPH, and 75% of women did not need further treatment after IUBT [2]. Nonetheless, IUBT has been mainly described after all deliveries including a quarter to half of cesarean deliveries in each sample of the largest published studies [8–12], and only one study with a very small sample (n = 66) has specifically analyzed the effectiveness of IUBT after vaginal delivery [13]. In a retrospective study in five maternity units in a perinatal network, we aimed to evaluate the effectiveness of IUBT specifically after vaginal delivery for management of persistent PPH, and to identify the risk factors of IUBT failure after vaginal delivery on this population.

## Material and methods

### Study sample

The study protocol and the consent procedure were approved by a Research Ethics Committee (*Comité d’Ethique de la Recherche en Obstétrique et Gynécologie*–CEROG—Paris) (N° 2016-OBST-03-28) on 30^th^ July 2016 before the beginning of the study. This study was conducted in accordance with the approved guidelines. All women received written information by the same midwife (MC). Written consent was not required for retrospective study according to the French law. This retrospective cohort study included all women who underwent IUBT, using Bakri balloon (Cook Medical, Bloomington, IN, USA), for persistent primary PPH after vaginal delivery from January 2011 to December 2015 in five maternity units in the same Pays de la Loire perinatal network in France. Postpartum hemorrhage (PPH) was defined by blood loss ≥500mL within 24 hours after birth. The Pays de la Loire perinatal network in France contains 23 maternities with more than 40,000 births/year. The five maternities were arbitrarily chosen because they have used more than fifteen Bakri balloons for persistent PPH after vaginal delivery during the study period. Three centers are tertiary public hospitals with more than 4,000 births/year, 3,500 births/year and 2,600 births/year and the two others are secondary private hospitals with 4,900 births/year and 3,000 births/year. Women were identified in the hospital discharge database by the procedure codes for “intrauterine balloon tamponade” and “postpartum hemorrhage”. An exclusion criterion was IUBT after cesarean delivery in the current pregnancy.

### Measures

The details of the procedures used to manage PPH after vaginal delivery, as well as maternal sociodemographic characteristics, information regarding pregnancy follow-up, clinical characteristics at admission, intrapartum variables and all clinical outcomes identified during the immediate postpartum period were retrospectively collected by the same midwife (MC).

All maternity belongs to the same perinatal network and all aspects of management of the third stage of the labor were identical in all maternity: intravenous injection of 5 IU oxytocin; placement of a graduated (100 mL graduation) collector bag [MVF Merivaara, Lay-St Christophe, France] just after birth which was left in place until the birth attendant judged that bleeding had stopped and that there was no reason to monitor further, and always at least for 15 minutes; suturing of lacerations; and manual removal of the placenta at 30 minutes after birth if not expelled. A common protocol for the stepwise management of PPH to stop bleeding after vaginal delivery was developed in all maternity units in the network, including both bimanual uterine massage, manual exploration of the uterus and administration of additional uterotonic agents such as sulprostone within 30 minutes of the PPH diagnosis if oxytocin fails to control the bleeding, and with IUBT if these actions failed to stop bleeding before recourse to either surgery or interventional radiology [14]. Nonetheless, the decision to use IUBT after vaginal delivery, using a Bakri balloon (Cook Medical, Bloomington, IN, USA), was left to the obstetrician’s discretion and was done with women’s agreement.

Maternal characteristics collected included age, pre-pregnancy body mass index (BMI, based on height and the first weight noted in the obstetric record), parity, previous cesarean section, and medical history, as history of PPH. Intrapartum variables recorded included gestational age at delivery determined by the craniocaudal length at a first-trimester ultrasound examination or by the date of last menstrual period and/or a second- or third-trimester ultrasound if the first-trimester ultrasound was not performed, as described in detail previously [15], prenatal suspicion of macrosomia determined by fundal height measurement at delivery > 37 cm and/or ultrasonographic fetal abdominal circumference > 90^th^ percentile for gestational age on Hadlock curves, as described in detail previously [16], detailed labor and delivery information, including type of labor (spontaneous or induced), mode of induction of labor (prostaglandins, amniotomy, or oxytocin), type of analgesia (intravenous, local, or regional), durations of labor (from 3cm to birth) and of the active phase of second stage (from the beginning of expulsive efforts to birth), soft tissue damage (perineal hematoma, third or fourth-degree perineal lacerations, cervical laceration), and birth weight.

The maternal short-term outcome considered included estimated blood loss measured with a collector bag [MVF Merivaara, Lay-St Christophe, France] placed just after birth [5,17], times from birth to PPH onset and to each stage of PPH management (sulprostone administration, balloon insertion), causes of PPH, need for manual removal of partial or total retained placenta, need for an additional uterotonic agent (i.e., sulprostone) due to PPH, mediolateral episiotomy (left to the discretion of the practitioner), soft tissue damage (perineal hematoma; second-, third- or fourth-degree perineal lacerations; cervical laceration), use of tranexamic acid, blood transfusions, invasive procedures for severe persistent PPH after failure of IUBT to stop the bleeding (defined by use of at least one of the following: uterine compression sutures, pelvic artery ligation, uterine embolization, or peripartum hysterectomy), and maternal postpartum complications, such as thromboembolic events (defined by a deep vein pulmonary embolism or thrombophlebitis), admission to intensive care, and maternal death. We also analyzed the characteristics of balloon introduction (volume inflated).

The objective was to determine predictors of failed IUBT in women with persistent PPH after vaginal delivery. The primary outcome was failure of IUBT, defined as a request of subsequent invasive procedures, such as pelvic arterial embolization, vessel ligation, uterine compression, or peripartum hysterectomy to stop bleeding. Women in the case group were defined as women who had IUBT but required another second line therapy (embolization or surgical procedures) to stop bleeding, and women whom IUBT stopped bleeding were defined as control group.

### Statistical analysis

A de-identified data set of women with IUBT was entered into EXCEL spreadsheets (S1 File), and migrated into the free Web statistics tool BiostaTGV software (https://marne.u707.jussieu.fr/biostatgv/) for analysis. No sample size calculation was done before the study and we collected data on all cases over a specified time period. Continuous parameters are given as means +/- standard deviation and compared by t-tests (or Mann-Whitney tests when appropriate); categorical variables were reported using counts and percentages and compared by chi-square tests (or Fisher exact tests when appropriate). Differences in maternal characteristics, obstetric, and PPH short-term-related outcomes were compared between the success and the failure groups. Crude odds ratio (OR) and 95% confidence interval (CI) were calculated for all factors studied in the analysis. Univariate and multivariable logistic regression analyses were conducted to determine independent predictors for failed IUBT. Factors identified as associated in univariate analysis with IUBT failure at a 0.2 level were included in this stepwise procedure. Statistical significance was defined as a *P* value < 0.05.

## Results

**Fig 1:**
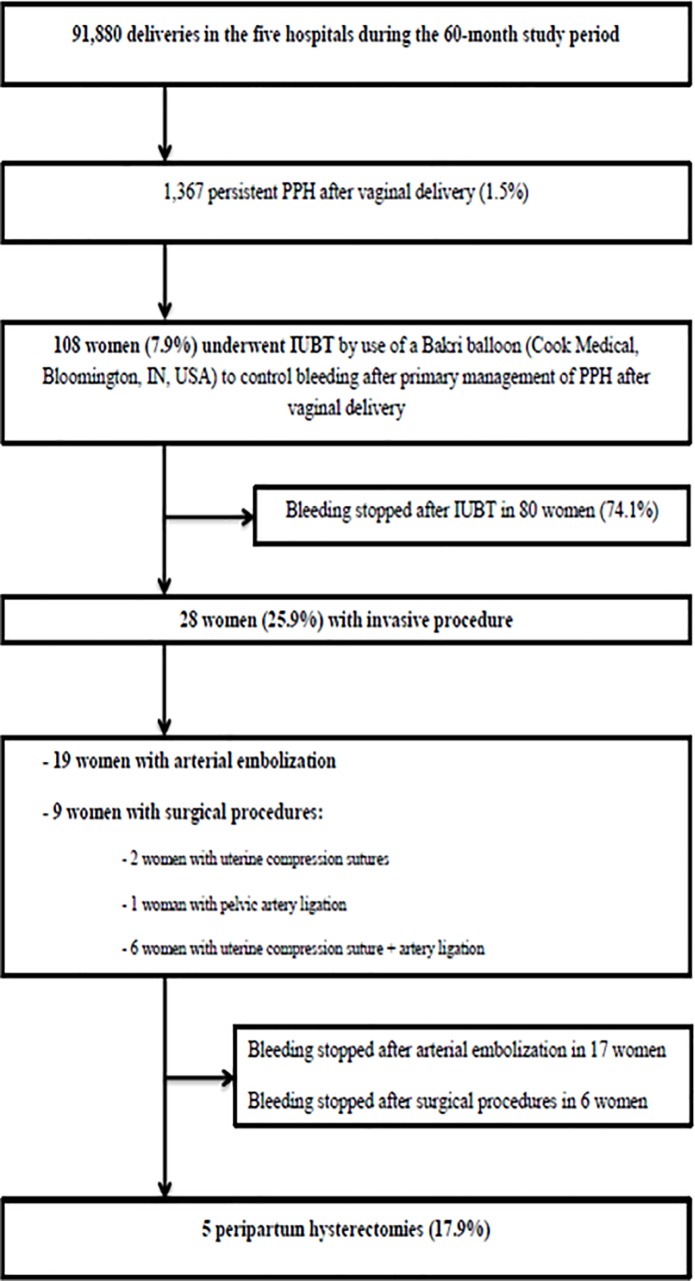
Cohort flowchart.

During the 60-month study period, there were 91,880 deliveries in the five hospitals, 1,367 persistent PPH after vaginal delivery (1.5%) and 108 women (7.9%) underwent IUBT by use of a Bakri balloon (Cook Medical, Bloomington, IN, USA) to control bleeding after primary management of PPH after vaginal delivery (Fig 1).

Table 1 summarizes the characteristics of the five maternities during the study-period (births, PPH, IUBT and failure of IUBT). Bleeding stopped after IUBT in 80 women (74.1%). In 28 women (25.9%), invasive procedures were required, including 19 arterial embolization and 9 surgical procedures with 5 peripartum hysterectomies (Fig 1). The rates of failure of IUBT (14.3–25.0%) were no significantly different according to the maternity (Table 1).

**Table 1:** Characteristics of the five maternities.

Maternity	Type	Births / study period	PPH / study period	IUBT	Weight (IUBT / PPH) x 100	Failure of IUBT
1	tertiary public hospital	13,363	1,170	16 (14.8)	1,4	7 (25.0)
2	secondary private hospital	16,056	557	21 (19.5)	3,8	6 (21.4)
3	tertiary public hospital	18,950	1,126	16 (14.8)	1,4	6 (21.4)
4	secondary private hospital	23,383	576	40 (37.0)	6,9	5 (17.9)
5	tertiary public hospital	20,128	1,479	15 (13.9)	1,0	4 (14.3)
**Total**		**91,880**	**4,908**	**108 (100)**	**2.2**	**28 (100)**

PPH: postpartum hemorrhage (defined by blood loss ≥500mL within 24 hours after birth); IUBT: intrauterine balloon tamponade

Failure of IBUT is defined as the performance of a subsequent invasive procedure, such as pelvic arterial embolization, vessel ligation, uterine compression, or peripartum hysterectomy to stop bleeding after balloon tamponade.

Table 2 detailed maternal and labor characteristics and maternal and neonatal outcomes according to IUBT efficiency. In the failure group, obese women (BMI≥30kg/m^2^) were significantly more frequent (25.9% compared to 6.1%; p = 0.03), and labor duration was significantly shorter (549.7±418.9 min compared to 363.9±314.0 min; p = 0.04). No other maternal or labor characteristics and neonatal outcome differed between groups (Table 2).

**Table 2:** Maternal characteristics, intrapartum variables and maternal and neonatal outcomes according to IUBT efficiency.

	Failure (n = 28)	Success (n = 80)	P *value*
**Maternal characteristics**			
Age (years)	31.7 ± 5.6	30.0 ± 5.1	0.13
Nulliparity, n (%)	8 (28.6)	29 (36.3)	0.46
Pre-pregnancy BMI (kg/m^2^)	24.7 ± 5.7	23.1 ± 4.0	0.11
BMI≥30 kg/m^2^ before pregnancy, n (%)	7 (25.9)	6 (7.5)	0.03
Previous cesarean section, n (%)	0 (0)	3 (3.8)	0.57
History of PPH, n (%)	6 (21.4)	19 (23.8)	0.80
History of hypertension, n (%)	2 (7.1)	0 (0)	0.07
Known uterine myoma, n (%)	0 (0)	1 (1.3)	1
Pregnancy obtained with ART, n (%)	4 (14.3)	10 (12.5)	0.75
Multiple pregnancy, n (%)	0 (0)	5 (6.2)	0.32
Hypertensive disease, n (%)	1 (3.6)	3 (3.8)	0.57
Gestational diabetes mellitus, n (%)	7 (25.0)	9 (11.3)	0.12
Anemia during pregnancy, n (%)	0 (0)	2 (2.5)	1
Antenatal suspicion of macrosomia, n (%)	1 (3.6)	7 (8.7)	0.68
**Intrapartum variables**			
Gestational age at delivery (weeks)	39.4±2.7	40.0 ± 1.7	0.33
Prolonged pregnancy (≥ 41 weeks)	8 (28.6)	24 (30.0)	0.86
Induced labor, n (%)	9 (32.1)	24 (30.0)	0.83
Labor duration (min)	363.9 ± 314.0	549.7 ± 418.9	0.04
Use of oxytocin, n (%)	14 (50.0)	51 (63.8)	0.20
Epidural analgesia, n (%)	21 (75.0)	69 (86.3)	0.24
Active phase of second stage longer than 30 min, n (%)	3 (10.7)	13 (16.3)	0.20
Spontaneous vaginal delivery, n (%)	20 (71.4)	57 (71.3)	1
Operative vaginal delivery, n (%)	8 (28.6)	23 (28.7)	1
**Maternal perineal outcome**			
Episiotomy, n (%)	5 (17.9)	20 (25.0)	0.41
Second-degree perineal laceration, n (%)	15 (53.5)	34 (42.5)	0.32
Third- or fourth-degree perineal laceration, n (%)	0	0	-
Perineal hematoma, n (%)	0	1 (1.7)	0.74
**Neonatal outcome**			
Birthweight (g)	3392.8 ± 784.1	3579.9 ± 493.4	0.24
Birthweight ≥ 4,000g	4 (14.3)	16 (20.0)	0.58

BMI: body mass index.

Data are mean ± standard deviations or n (%) or unless otherwise specified. Student t test, χ^2^ test, non-parametric Mann-Whitney test, and Fisher’s exact test were used as appropriate.

Uterine atony (48.1% (13/28) compared to 44.8% (26/80); p = 0.08) was more frequent in the failure group. The success and failure groups had similar rates of active management of PPH (diagnosis of PPH period, management, sulprostone treatment, and estimated blood loss) (Table 3). Estimated blood loss at balloon insertion was higher in the failure group (1785.4±697.3 mL compared to 1434.6±540.0mL; p = 0.01) and PPH ≥ 1,500 mL before use of IUBT was more frequent in the failure group (64% compared to 40%; p = 0.04). Nonetheless, the mean time from birth to IUBT (109.7±95.3 compared to 114.1±66.1min; p = 0.82) did not differ between groups (Table 3).

**Table 3:** Causes, management and outcome of PPH according to intrauterine balloon tamponade efficiency.

	Failure (n = 28)	Success (n = 80)	P *value*
Uterine atony, n (%)	13 (46.4)	26 (32.5)	0.20
Placenta praevia, n (%)	1 (3.6)	5 (6.2)	0.67
Placenta accrete, n (%)	2 (7.2)	1 (1.3)	0.18
Retained placenta (total or partial), n (%)	6 (21.4)	17 (21.3)	0.97
Time from birth to PPH onset (min)	36.7 ± 37.8	43.0 ± 75.0	0.59
Estimated blood loss at PPH onset (mL)	687.4 ± 271.4	605.3 ± 267.4	0.21
Manual removal of retained placenta, n (%)	9 (32.1)	21 (26.3)	0.85
Use of tranexamic acid, n (%)	19 (67.9)	35 (43.8)	0.03
Need for sulprostone, n (%)	28 (100)	80 (100)	-
Time from birth to sulprostone, n (%)	72.1 ± 50.6	71.8 ± 84.0	0.98
Time from birth to IUBT (min)	114.1 ± 66.1	109.7 ± 95.3	0.82
Estimated blood loss at balloon insertion (mL)	1,785.4 ± 697.3	1,434.6 ± 540.0	0.01
Estimated blood loss at balloon insertion ≥ 1,500 mL, n (%)	19 (67.9)	38 (47.5)	0.04
Volume inflated of the balloon (cc)	458.8 ± 48.1	420.2 ± 103.1	0.06
Estimated blood loss after balloon placement (mL)	1,195.0 ± 182.3	341.0 ± 112.2	<0.0001
Time from birth to stop bleeding (min)	309.4 ± 145.2	208.4 ± 136.4	0.002
Total estimated blood loss when bleeding had stopped (mL)	2,980.4 ± 879.6	1,775.6 ± 652.2	<0.0001
Red blood cells transfusion, n (%)	8 (28.6)	10 (12.5)	0.03
Blood transfusion ≥ 4 packed red blood cells, n (%)	26 (92.9)	25 (31.3)	<0.0001
Fresh frozen plasma transfusion, n (%)	8 (28.6)	8 (10.0)	0.02
Platelets transfusion, n (%)	4 (14.3)	4 (5.0)	0.20
Fibrinogen concentrates, n (%)	8 (28.6)	7 (8.8)	0.01
Activated factor VII, n (%)	3 (10.7)	0	0.08
Thromboembolic event, n (%)	0	2 (2.5)	0.55
Admission to intensive care unit, n (%)	18 (64.3)	8 (10)	<0.0001
Length of stay in intensive care unit (days)	1.8 ± 1.3	2.9 ± 4.2	0.49
Total length of stay in maternity (days)	6.1 ± 2.0	4.8 ± 1.9	0.007
Maternal death, n (%)	0	0	-

IUBT: intrauterine balloon tamponade.

Data are mean ± standard deviations or n (%) or unless otherwise specified. Student t test, χ^2^ test, non-parametric Mann-Whitney test, and Fisher’s exact test were used as appropriate.

Mean estimated blood loss after balloon placement (1195.0±182.3mL compared to 341.0±112.2mL; p<0.0001) and mean elapse time of bleeding stop from birth (309.4±145.2min compared to 208.4±136.4min; p = 0.002) were significantly higher in the failure group. As consequences, women in the failure group had higher total blood loss (2,980.4±879.6mL compared to 1,775.6±652.2mL; p<0.0001). Frequencies of use of tranexamic acid, and packed red blood cells, fresh frozen plasma and fibrinogen concentrates transfusions, and transfusion of ≥4 U of packed red blood cells were significantly higher in the failure group (Table 3), in accordance with the perinatal network guidelines of management of persistent PPH which recommended systematic transfusions and use of tranexamic acid in these cases. The women who underwent intensive care units admission after delivery were more frequent (64.3% compared to 10.0%; p<0.0001) in the failure group, and had longer hospitalizations in the maternity unit (6.1±2.0 compared to 4.8±1.9 days; p = 0.007) (Table 3). Overall, only two women, who were in the success group, had postpartum complications (thromboembolic events). There was no maternal death.

After adjustment for confounding factors in the multivariate logistic regression analysis (maternal age, obesity, labor duration and estimated blood loss at balloon insertion), obesity was a risk factor of failed IUBT (aOR 4.40, CI95% 1.06–18.31). Major PPH (≥1,500mL) at balloon insertion seemed to be another risk factor of failure (aOR 1.001, CI95% 1.000–1.002), but our result did not reach statistical significativity.

## Discussion

### Main findings

This retrospective cohort study confirmed the effectiveness of IUBT in our perinatal network with a high rate of success of this process (74.1%) to stop bleeding after primary management of PPH after vaginal delivery. We also demonstrated that pre-pregnancy obesity and major PPH (≥1,500mL) at balloon insertion were risk factors of failed IUBT.

### Interpretation

Our results are consistent with previous studies that reported rates of success of IUBT from 60 to 94% [8,10,18,19], and a systematic review from 46 studies reported an overall rate of success of 84% for IUBT [18]. Moreover, some authors have suggested that IUBT may be an effective solution of stabilizing a woman with persistent PPH before maternal transfer to an embolization unit [14] or prevent the need for embolization or surgery [20]. Nonetheless, IUBT has been mainly described after all deliveries including a quarter to half of cesarean deliveries in each sample of the largest published studies [8–12], and only one study has specifically analyzed the effectiveness of IUBT after vaginal delivery [13]. Recently, Darwish et al. [13] reported a single blinded randomized controlled trial conducted in Egypt including 66 women with primary atonic PPH following vaginal delivery to assess the efficacy of condom-loaded Foley's catheter versus Bakri balloon. In this small size sample study, Bakri balloon was an effective tool (30/33; 91.0%) for the management of primary atonic PPH following vaginal delivery [13].

In our study, pre-pregnancy obesity was a risk factor of failed IUBT (aOR 4.40, 95% CI 1.06–18.31), that has never been reported. It’s an interesting result for physician because obesity is well known as a risk factor of PPH [21], and it appears to be also a risk factor of failure of IUBT. The information gained from this study is helpful in counselling physicians; obese women with persistent PPH after vaginal delivery are high-risk women for failed IUBT and will probably require more often invasive procedure to achieve bleeding after vaginal delivery and to improve maternal outcomes.

We reported that the mean time from birth to IUBT (109.7±95.3 compared to 114.1±66.1min; p = 0.82) did not differ between groups and between maternity. Nonetheless, estimated blood loss at balloon insertion was higher in the failure group (1785.4±697.3 mL compared to 1434.6±540.0mL; p = 0.01) and major PPH (≥1,500 mL) before use of IUBT was more frequent in the failure group (64% compared to 40%; p = 0.04). Major blood loss (≥1,500mL) before IUBT seems to be another predictive factor of failure (aOR 1.001, 95% CI 1.000–1.002; p = 0.055), but our result did not reach statistical significativity. These results are in concordance with Howard et al. [22] who analyzed the efficacy of IUBT to decrease the maternal morbidity in 420 women with PPH. They showed that women receiving IUBT at lower estimated blood loss quartiles had with decreased maternal morbidity with higher nadir hemoglobin, less frequent packed red blood cell transfusion, fewer intensive care unit admissions, and fewer hysterectomies. The risk of coagulopathy increases quickly with blood loss and early IUBT may prevent its occurrence. Vintejoux et al. [9] also showed a 100% success rate of IUBT in case of early use (defined by a blood loss less than 1,000mL). Recently, a prospective cohort study in ten maternity units in a perinatal network in France including 226 women with IUBT (171 after vaginal delivery and 55 during or after caesarean delivery) showed that estimated blood loss before IUBT (>1,500mL) was a predictive factor of IUBT failure (aOR 3.2, 95% CI 1.5–6.8) [10]. Our study was probably underpowered to show a same significant result but these results may reinforce the idea that IUBT must be used earlier in the management of persistent PPH.

### Strengths and limitations

The principal strength of this study is that we only included women who required IUBT for a persistent PPH after vaginal delivery in a large retrospective multicentre study to identify the factors predicting IUBT failure. Most of the largest published studies mainly described the use of IUBT after all deliveries including a quarter to half of cesarean deliveries [8–12] that may modify analysis and conclusions. Second, all maternity units in the network followed a common protocol for stepwise management of PPH after vaginal delivery, with IUBT required as the initial second-line therapy, even though the decision to use IUBT after vaginal delivery was left to the obstetrician’s discretion.

The main limitation is the retrospective design of this study limiting conclusions. Results may be biases from unknown factors that have been not collected in our study, even though exhaustive data were collected for all important factors potentially associated with IUBT failure (maternal characteristics, intrapartum variables such as type of labor or estimated blood loss before IUBT insertion, timing of the different step of PPH management). Second, we have arbitrarily chosen five maternities of the perinatal network which usually used IUBT with Bakri balloon (more than 15 cases) during the study period. That may introduce a selection bias about the results, even though we have selected maternities with the most trained teams about IUBT with Bakri balloon. Third, no formal sample size was calculated as we collected data on all cases over a specified time period. This means that some characteristics that might be truly predictive of failure may not have been identified due to a type 2 error.

## Conclusion

We found that intrauterine balloon tamponade using Bakri balloon is an effective second line therapy for persistent primary PPH after vaginal delivery. Using IUBT as a systematic part of the management is a reasonable addition to PPH protocols. Maternal obesity at Bakri balloon insertion seemed to be a risk factor of failure of the device, and when Bakri balloon is placed late for persistent PPH after vaginal delivery that it is less likely to be successful. Furthermore, additional prospective studies included numerous women with persistent PPH after vaginal delivery are needed to confirm these results and to test the safety of IUBT to reduce maternal morbidity and mortality.

## Supporting information

S1 File A de-identified data set of women with IUBT. (XLSX)
